# Acute Bilateral Visual Loss as an Atypical Manifestation of Wernicke Encephalopathy Secondary to Hyperemesis Gravidarum: A Case Report and Narrative Literature Review

**DOI:** 10.7759/cureus.115540

**Published:** 2026-08-31

**Authors:** Law Ngee Ling, Siti Nur Amira Abu Kassim, Juliana Jalaluddin, Ayesha Mohd Zain

**Affiliations:** 1 Ophthalmology, University Kebangsaan Malaysia Medical Centre, Kuala Lumpur, MYS; 2 Ophthalmology, Hospital Melaka, Melaka, MYS

**Keywords:** hyperemesis gravidarum, pregnancy complication, thiamine nutritional deficiency, transient blindness, wernicke encephalopathy

## Abstract

Wernicke encephalopathy (WE) is a severe neurological condition caused by thiamine deficiency that remains underreported in pregnancy. We report a clinical diagnosis of WE in a 41-year-old woman, gravida 2, para 1, at 16 weeks of gestation. She presented with acute, painless visual impairment, severe dietary restriction, and marked weight loss secondary to severe hyperemesis gravidarum (HG). Examination revealed reduced visual acuity, horizontal nystagmus, psychomotor retardation, and an ataxic gait. The initial diagnosis was delayed due to her atypical presentation and the absence of MRI or blood thiamine level confirmation. However, empirical treatment with IV thiamine was followed by rapid visual and cognitive recovery within 24 hours. The pregnancy was subsequently complicated by fetal growth restriction and preterm delivery. This case illustrates the necessity of empirical treatment for WE based on clinical criteria in pregnant patients with HG. Recognizing atypical presentations and administering thiamine promptly are critical because delayed treatment may result in irreversible maternal neurological complications and adverse pregnancy outcomes.

## Introduction

Wernicke encephalopathy (WE) is a severe neurological condition characterized by thiamine deficiency [1]. Thiamine acts as an essential coenzyme in cerebral energy metabolism, and its rapid depletion leads to neuronal damage in regions of the brain with high metabolic demands, including the medial thalamus, mammillary bodies, and oculomotor nuclei [2-4]. While classically associated with alcohol misuse, WE is increasingly recognized in non-alcoholic populations [5]. Pregnant women experiencing hyperemesis gravidarum (HG), a condition characterized by intractable nausea and vomiting, are at high risk due to prolonged dietary restriction, recurrent gastrointestinal losses, and the increased metabolic demands associated with gestation [5,6]. A systematic review by Oudman E et al. (2019) found that WE remains underreported and is frequently misdiagnosed in the obstetric setting [6].

The classic diagnostic triad of confusion, ataxia, and ophthalmoplegia is observed in only a minority of patients [2,6,7]. Typically, ophthalmic manifestations of WE arise from lesions in the brainstem oculomotor nuclei, presenting as nystagmus and lateral rectus or conjugate gaze palsies [8]. Therefore, acute profound bilateral visual loss without classical ophthalmoplegia is an atypical presentation, as it implies primary involvement of the optic nerve or retinal ganglion cells rather than the typical extraocular motor pathways. This distinction has clinical implications; isolated visual impairment can be misattributed to other causes of acute or subacute bilateral visual loss in pregnancy. For instance, vitamin B12 deficiency can also induce optic neuropathy; however, it typically develops subacutely over months. This contrasts with the rapid, acute visual deterioration seen in acute thiamine depletion.

Atypical presentations often delay diagnosis. Delayed administration of thiamine increases the risk of irreversible maternal neurological complications, such as Korsakoff’s syndrome, and adverse pregnancy outcomes, including fetal loss [2,3,6]. To facilitate earlier recognition, clinicians can apply the Caine criteria, which require the presence of at least two of four signs: dietary deficiency, oculomotor abnormalities, cerebellar dysfunction, and altered mental state [7]. However, these criteria were validated in chronic alcoholic populations; thus, in the obstetric setting, they serve as a supportive clinical aid to guide early empirical intervention rather than as a definitive diagnostic test.

While the clinical reversal of WE is well documented, objective structural tracking of WE-associated optic neuropathy over time is rarely reported in the obstetric literature. This report addresses this gap by corroborating recently emerging evidence [8], presenting a case of WE secondary to HG featuring atypical, profound visual loss, and utilizing longitudinal optical coherence tomography (OCT) to objectively document the evolution of optic nerve changes. Ultimately, this case underscores the educational value of recognizing atypical neuro-ophthalmic presentations and reinforces the necessity of prompt empirical clinical management.

## Case presentation

A 41-year-old woman, gravida 2, para 1, at 16 weeks of gestation, presented with generalized, painless blurred vision of two weeks’ duration, with no other ocular complaints. Her medical history included hospitalization one month prior for HG, characterized by severe anorexia and a 15 kg weight loss over two months. Since week 8 of gestation, she had been experiencing daily vomiting, which severely limited her diet to minimal amounts of porridge.

Examination revealed a cachectic state and delayed cognitive responses, with stable vital signs. Neurological examination showed normal motor strength but an ataxic gait. Aided visual acuity, measured using a Snellen chart, with refraction revealing bilateral low myopic astigmatism, was limited to hand movements in the right eye and counting fingers in the left eye. Small-magnitude horizontal nystagmus was noted, with otherwise full extraocular motility. Pupillary examination revealed bilateral, equal, and reactive pupils with no relative afferent pupillary defect (RAPD). Intraocular pressure was within normal limits bilaterally. Confrontation visual field, formal color vision, contrast sensitivity, and automated perimetry assessments were precluded by the profound severity of the visual impairment. Electrophysiological investigations, such as visual evoked potentials (VEP), were not performed due to institutional facility limitations. A fetal scan showed normal fetal parameters.

The anterior segment examination and macular OCT were unremarkable. While the peripheral retina and macula appeared normal, the optic nerve head demonstrated minimal temporal optic disc edema, evidenced by localized thickening of the temporal retinal nerve fiber layer (RNFL) (Figure 1). Quantitatively, the initial global average RNFL thicknesses were elevated (115 µm in the right eye and 121 µm in the left eye).

**Figure 1:**
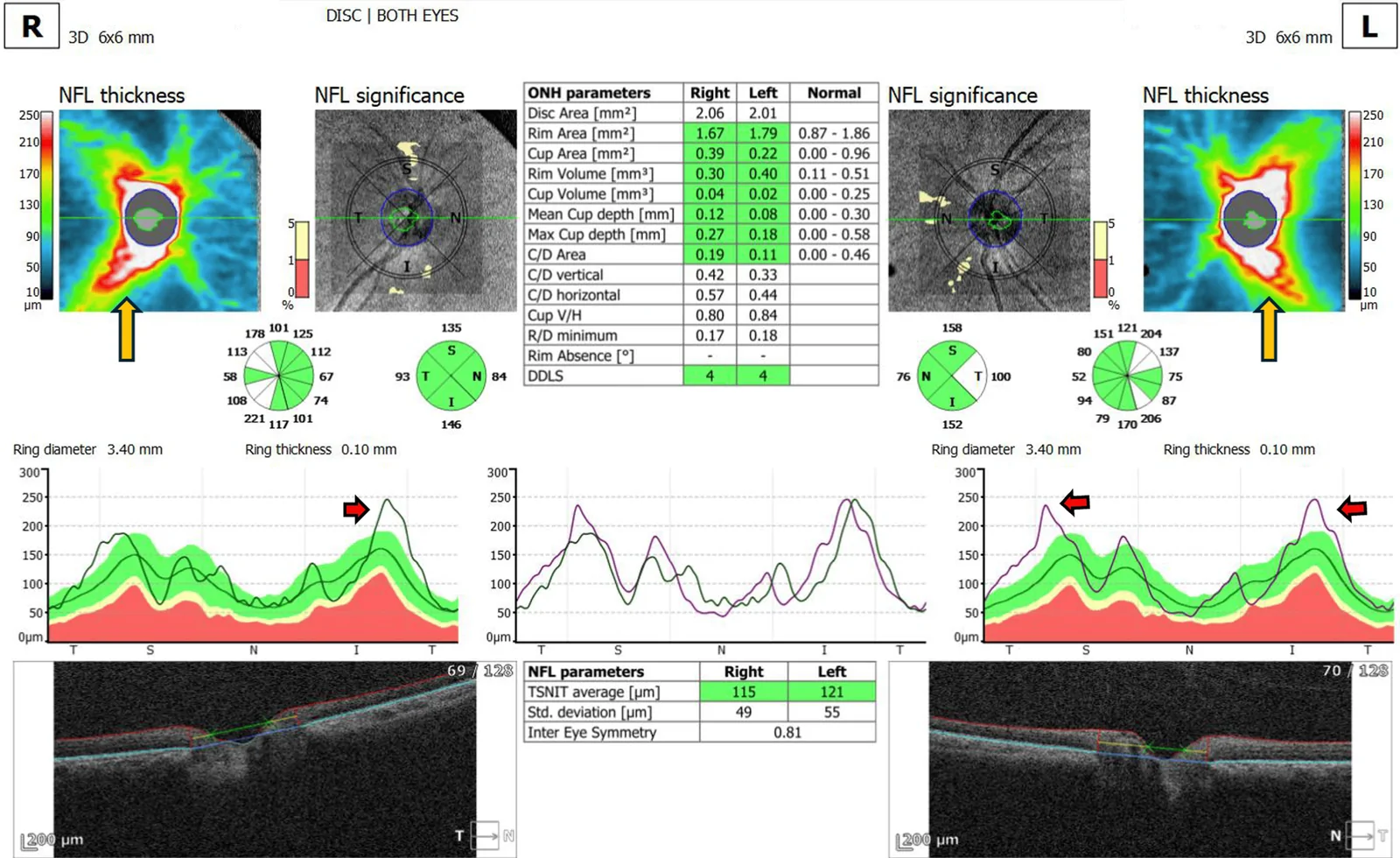
Initial OCT peripapillary RNFL assessment. The native printout displays quantitative measurements, scan parameters (3D 6 × 6 mm), and laterality for the right (R) and left (L) eyes. On the RNFL thickness maps (top), the yellow arrows indicate localized areas of thickening in the temporal and inferior-temporal regions, visually represented by the white and red color mapping. Correspondingly, on the temporal-superior-nasal-inferior-temporal (TSNIT) graph, the red arrows highlight the patient’s RNFL thickness profile extending above the normal green band (5th-95th percentile) and into the white supranormal zone (>95th percentile). These findings are consistent with acute temporal optic disc edema without evidence of inner retinal disruption. RNFL: Retinal nerve fiber layer; R: Right eye; L: Left eye; TSNIT: Temporal-superior-nasal-inferior-temporal; OCT: Optical coherence tomography; mm: Millimeter(s).

MRI of the brain was not immediately available during the acute presentation; thus, an urgent plain CT scan of the brain was performed as an alternative and revealed no abnormalities. Laboratory tests indicated mild anemia (hemoglobin: 10.8 g/dL (normal: 13.0-17.0 g/dL)), hyponatremia (sodium: 132 mmol/L (normal: 136-145 mmol/L)), elevated urea (urea: 9.8 mmol/L (normal: 2.76-8.07 mmol/L)), impaired renal function (eGFR: 51.6 mL/min/1.73 m² (normal: >90 mL/min/1.73 m²)), and low vitamin B12 (73.6 pmol/L (normal: 145-569 pmol/L)). Other parameters, such as serum calcium, magnesium, phosphate, iron, and serum folate, were normal.

Due to the atypical nature of her painless visual loss without classic ophthalmoplegia, her presentation initially confounded frontline medical practitioners. She remained in the ED for over 48 hours, undergoing extensive investigations across multiple disciplines, including ophthalmology, obstetrics, neurology, and internal medicine. During the initial 48 hours of her emergency admission, she received IV 5% dextrose-normal saline (NSD5) without concurrent thiamine supplementation while undergoing diagnostic evaluation. This prolonged diagnostic phase caused a critical delay in the initiation of targeted therapy. Upon clinical suspicion of WE, she was immediately transitioned to high-dose intravenous thiamine therapy. The regimen commenced with IV thiamine 500 mg three times daily for two days, followed by 200 mg three times daily for three days. Hyperemesis was controlled with intravenous metoclopramide 10 mg three times daily. Her vision improved within hours, achieving an aided visual acuity of 6/6 on a Snellen chart within 24 hours. Her cognition and alertness markedly improved, and her acute kidney injury resolved with hydration. Repeat serum vitamin B12 levels after treatment normalized (561 pmol/L). She was discharged with oral thiamine, which was tapered weekly from 100 mg daily for one week, followed by 50 mg, 40 mg, 30 mg, and 20 mg daily for one week each, and maintained at 10 mg daily throughout the remainder of her pregnancy.

At the 6-month follow-up, the patient maintained a visual acuity of 6/6 bilaterally. Repeat peripapillary OCT revealed resolution of the acute optic disc swelling; however, it demonstrated subsequent temporal and inferior RNFL thinning. Quantitatively, while the global average RNFL thicknesses remained within normal limits (81 µm in the right eye and 83 µm in the left eye), the temporal-superior-nasal-inferior-temporal (TSNIT) profile demonstrated focal downward deviation into the borderline (yellow zone, <5th percentile) and abnormal ranges (red zone, <1st percentile), specifically within the temporal and inferior segments compared with the age-matched normative database (green zone, 5th-95th percentile) (Figure 2).

**Figure 2:**
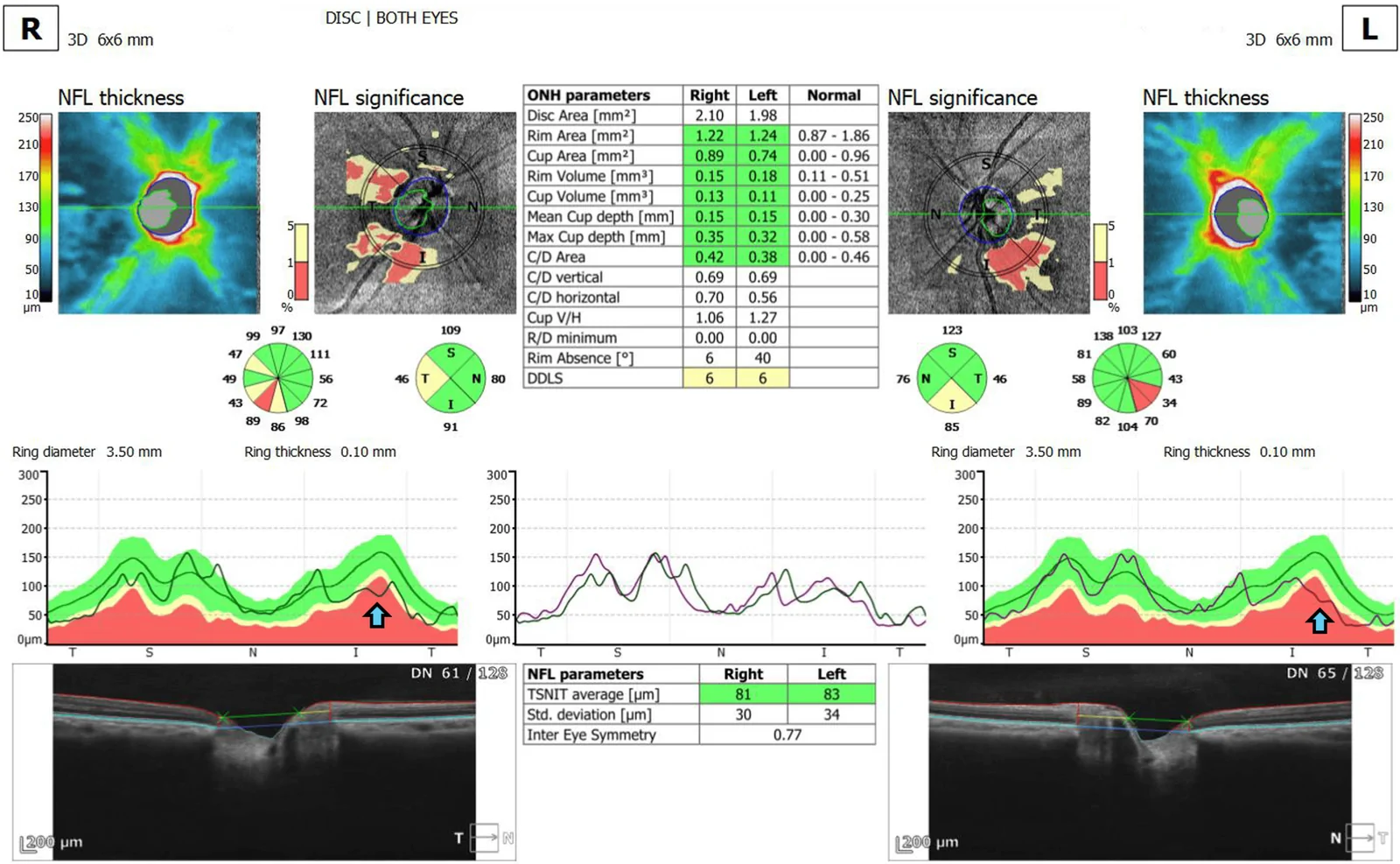
Follow-up bilateral OCT peripapillary RNFL assessment at six months. Serial follow-up OCT scans demonstrate resolution of the acute optic disc swelling. The blue arrows highlight focal downward deviations into the red abnormal zone (<1st percentile) on the TSNIT graphs, indicating subsequent temporal and inferior RNFL thinning. Compared with baseline, these serial scans illustrate the structural evolution from acute edema to subclinical RNFL thinning over time. OCT: Optical coherence tomography; RNFL: Retinal nerve fiber layer; TSNIT: Temporal-superior-nasal-inferior-temporal.

The infant was delivered preterm at 34 weeks with a birth weight of 1.39 kg via emergency lower segment cesarean section due to abnormal cardiotocography and intrauterine growth restriction (IUGR). Given the patient’s history of severe HG and general maternal malnutrition, these obstetric complications were likely multifactorial. A direct causal relationship between these fetal outcomes and WE cannot be definitively established. Detailed antenatal growth trajectories and long-term pediatric neurodevelopmental follow-up data were unavailable in our clinical records; however, the infant was noted to be healthy at 2 months of age. Clinical screening for retinopathy of prematurity (ROP) revealed a normal retinal examination, and the infant was discharged from the ophthalmology clinic. The chronological course of the patient’s clinical presentation, treatment, follow-up, and pregnancy outcome is summarized in Figure 3.

**Figure 3:**
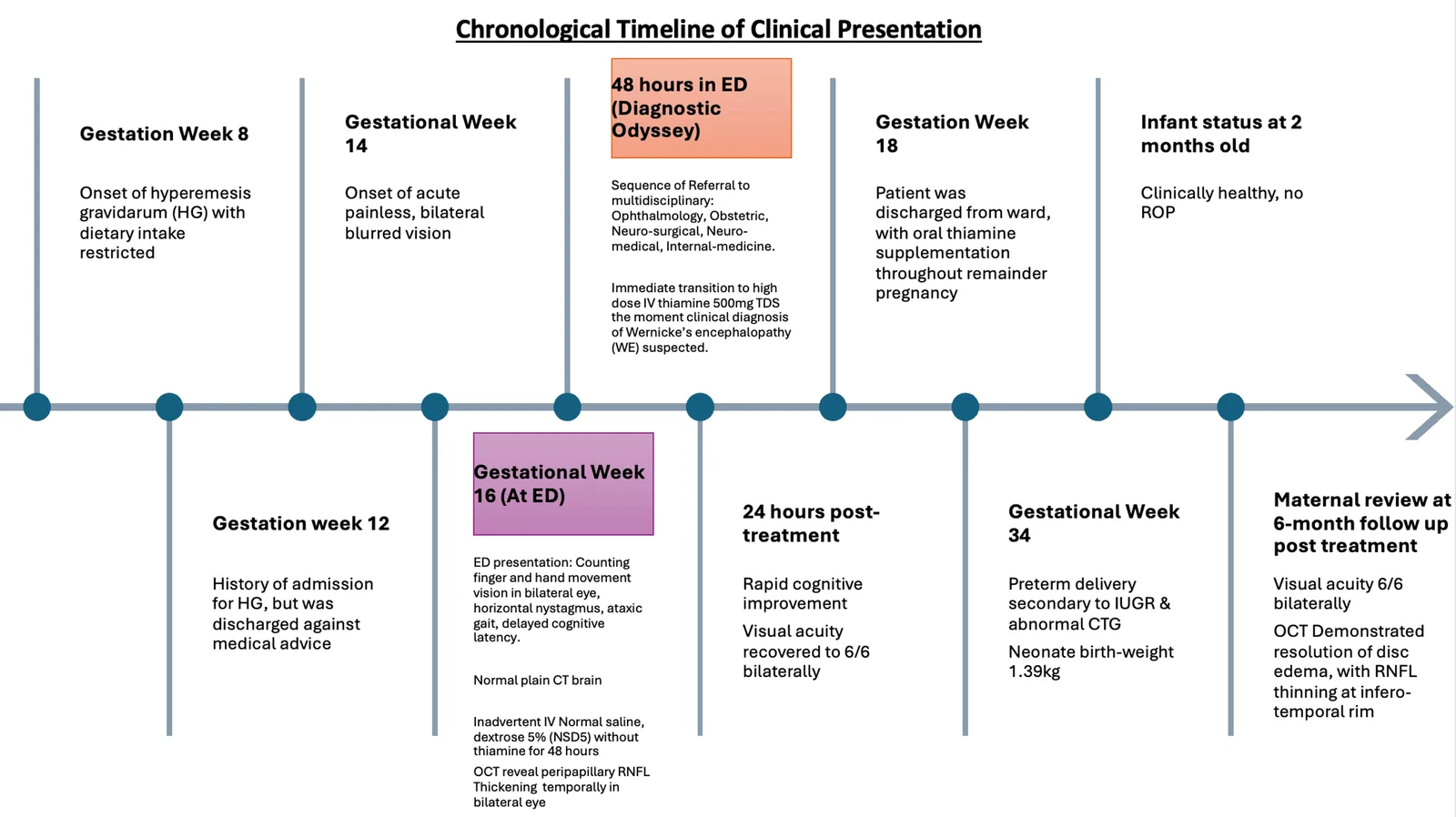
Chronological timeline of clinical presentation. HG: Hyperemesis gravidarum; OCT: Optical coherence tomography; CTG: Cardiotocography; RNFL: Retinal nerve fiber layer; WE: Wernicke encephalopathy; ROP: Retinopathy of prematurity.

## Discussion

This case demonstrates the importance of maintaining a high index of suspicion for WE in pregnant patients with severe HG. Early diagnosis of WE is challenged by its nonspecific symptoms, which can include mental status changes, ocular abnormalities, and ataxia [2]. Our patient's atypical presentation with visual disturbances further supports the need for heightened awareness in pregnant women presenting with severe nausea and vomiting.

To contextualize our findings, a narrative literature review was conducted to evaluate atypical presentations and clinical outcomes of WE in pregnancy. A search of the PubMed and Google Scholar databases was performed for English-language articles published up to 2025, using the primary search terms “Wernicke Encephalopathy,” “hyperemesis gravidarum,” and “visual loss.” Articles were selected based on their relevance to atypical ophthalmic presentations, diagnostic delays, and maternal-fetal outcomes. These cases and reviews are summarized in Table 1.

**Table 1:** Summary of selected published literature on Wernicke encephalopathy in pregnancy and hyperemesis gravidarum. WE: Wernicke encephalopathy; HG: Hyperemesis gravidarum; IV: Intravenous; IM: Intramuscular; OCT: Optical coherence tomography; RNFL: Retinal nerve fiber layer.

Author and Year	Study Type	Key Clinical and Diagnostic Findings	Treatment, Outcomes, and Implications
Rees JH et al. (1997) [1]	Case series (n=2)	Symptoms included confusion, ataxia, and diplopia. IV carbohydrates without thiamine triggered neurological decline.	One case resulted in fetal death. Emphasizes administering thiamine before or alongside IV fluids.
Wilson RK et al. (2006) [5]	Case report (n=1)	Presented with HG, confusion, papilledema, and hearing loss. Brain MRI showed bilateral medial thalamic hyperintensities.	Fetal loss preceded maternal focal signs. Highlights the high risk of WE in non-alcoholic, malnourished populations.
Oudman E et al. (2019) [6]	Systematic review (n=177)	The classic triad was absent in 37.9% of WE cases in HG. Visual symptoms (double/blurred vision) were common prodromes.	Fetal loss occurred in 50% of evaluated pregnancies (71/142 cases); maternal death occurred in 5% (9/177 cases). Recommends 100 mg prophylactic IV/IM thiamine for severe HG.
Palakkuzhiyil N et al. (2019) [9]	Case report (n=1)	WE presented atypically with reduced vision, papillitis, and gaze-evoked nystagmus. MRI showed periaqueductal hyperintensities.	Visual loss and optic neuropathy resolved rapidly after 500 mg IV thiamine.
Divya MB et al. (2022) [2]	Case series (n=6)	Atypical signs included slurred speech, visual loss, and seizures. Brain MRI scans were completely normal in 50% of patients.	Immediate parenteral thiamine yielded neurological recovery in all patients. Relying on MRI delays life-saving empirical treatment.
Cetera GE et al. (2023) [4]	Case report (n=1)	Presented with dizziness, memory loss, and absent deep tendon reflexes. WE was compounded by pre-gestational hyperthyroidism.	Prompt thiamine allowed for a healthy term delivery. Mild anterograde amnesia persisted despite treatment.
Pham A et al. (2023) [3]	Case report (n=1)	Severe encephalopathy and ophthalmoplegia following HG. MRI revealed thalamic and mammillary body hyperintensities.	Resulted in intrauterine fetal demise. Highlights that untreated or inadequately treated WE leads to permanent Korsakoff syndrome in ~80% of cases (foundational literature establishes this exact rate at 84% [10]).
Parrish C et al. (2024) [11]	Clinical review (n=N/A)	Clinicians often have a low index of suspicion. Whole-blood thiamine assays take 7-10 days to process.	Treatment must be empirical; never wait for laboratory confirmation.
Bensaid A et al. (2025) [12]	Case report (n=1)	Ataxia, nystagmus, and hyperlactatemia with severe pre-eclampsia. Initial CT was normal; MRI confirmed the diagnosis.	Immediate IV thiamine administration led to full maternal recovery. MRI remains the gold standard for WE diagnosis. (While the authors erroneously cite a 93% sensitivity for MRI, foundational neuroimaging literature establishes MRI specificity at 93% and sensitivity at ~53% [13].)
Kal M et al. (2025) [8]	Clinical report (n=1)	Utilized swept-source OCT to diagnose and monitor optic neuropathy in WE secondary to HG. Documented the transition from acute optic disc edema to focal RNFL thinning.	Structural deficits (RNFL thinning) can persist despite functional visual recovery. Validates OCT as an objective tool for longitudinal monitoring in atypical WE.

Recent studies note the prevalence of WE in non-alcoholic populations, particularly in relation to HG [5]. The demand for thiamine during pregnancy increases due to heightened metabolic needs, compounded by the rapid depletion of stores from persistent vomiting [2,6]. Palakkuzhiyil N et al. (2019) documented a closely mirroring case of WE with visual loss in HG [9]. Our case illustrates this dynamic, showing significant weight loss correlating with inadequate dietary thiamine. Serum thiamine levels were omitted in the acute setting because whole-blood thiamine assays require 7 to 10 days to process, which delays the initiation of empirical parenteral thiamine [11]. Furthermore, serum thiamine lacks sensitivity and specificity, and no universally established laboratory value exists to conclusively diagnose WE [11].

WE remains frequently missed because the classic triad of confusion, ophthalmoplegia, and ataxia is not always present [2-4,6,11]. The lack of the obvious classical triad initially confounded the primary care team, resulting in a 48-hour diagnostic delay in the emergency department prior to thiamine initiation. Therefore, healthcare providers, especially those working in obstetrics and primary care, must be educated about WE and its atypical presentations to avoid detrimental delays. In the absence of the classical triad, clinical suspicion can be guided by the Caine criteria. These criteria require the presence of at least two of four signs: dietary deficiencies, oculomotor abnormalities, cerebellar dysfunction, and an altered mental state or mild memory impairment [7]. While these criteria demonstrated an 85% diagnostic sensitivity in their original validation study, this figure was established specifically in chronic alcoholic populations [7]. These criteria are widely extrapolated as supportive clinical tools in non-alcoholic populations [14], including those with HG, to justify empirical intervention rather than serving as a definitive diagnostic test. Retrospectively, our patient fulfilled three of these supportive criteria upon initial presentation. Her prolonged hyperemesis and 15 kg weight loss satisfied the requirement for severe dietary deficiency; her ataxic gait confirmed cerebellar dysfunction; and her delayed cognitive latency with psychomotor retardation, representing the profound apathy and cognitive blunting characteristic of early encephalopathy, fulfilled the criterion for an altered mental state. Had these criteria been recognized and applied prospectively, they could have facilitated an immediate clinical diagnosis, effectively avoiding her 48-hour diagnostic delay.

A clinical finding that may confound practitioners in atypical cases is the absence of a RAPD despite profound bilateral visual loss. An RAPD should be present in unilateral optic neuropathy but can be absent when the optic neuropathy is bilateral and symmetrical, in which case the test cannot be used diagnostically [15]. This phenomenon is characteristic of relatively symmetrical, bilateral optic neuropathies caused by nutritional deficiencies or toxic etiologies [16]. Optic neuritis was clinically excluded, as it typically presents with unilateral, painful visual loss exacerbated by eye movement in 90% of patients [17], whereas our patient experienced painless, bilateral impairment. Bilateral ischemic optic neuropathy was unlikely given the absence of severe acute hemodynamic compromise or malignant pre-eclampsia, and ischemic damage does not reverse within 24 hours of vitamin supplementation [18]. Intracranial hypertension and posterior reversible encephalopathy syndrome (PRES) were clinically improbable due to the absence of severe headaches, seizures, malignant hypertension, or severe papilledema [19]. The normal brain CT scan made major structural and vascular etiologies, such as cerebral venous sinus thrombosis (CVST), less likely [20]. However, early CT can be normal in occipital infarction, which may require MRI as a definitive test [21]. While toxic, metabolic, or severe electrolyte disturbances can alter mental status, they rarely cause isolated, profound acute blindness because nutritional and toxic optic neuropathies characteristically produce progressive rather than abrupt visual loss [16].

The initial laboratory tests revealed a markedly low vitamin B12 concentration, raising the clinical possibility of a concurrent nutritional optic neuropathy. However, total serum vitamin B12 levels frequently demonstrate a physiological decline during normal pregnancy. This is primarily ascribed to progressive plasma volume expansion (hemodilution) and a pregnancy-induced reduction in haptocorrin, a primary B12-binding protein [22]. These physiological shifts can cause spuriously low total B12 readings while the active tissue form (holotranscobalamin) remains stable, meaning functional tissue depletion is absent [22]. Furthermore, B12-induced optic neuropathy typically presents subacutely or chronically over several months, contrasting with the acute two-week decline observed here. Our patient achieved complete visual and cognitive recovery within 24 hours of receiving isolated intravenous thiamine, without vitamin B12 replacement therapy, clinically supporting acute thiamine depletion as the primary etiology.

The longitudinal OCT documentation provides structural evidence tracking the evolution of WE-associated optic neuropathy in the setting of HG. In alignment with recent findings by Kal M et al. (2025) [8], our serial scans demonstrate a distinct transition from acute temporal optic disc edema to focal temporal and inferior RNFL thinning at the 6-month follow-up, despite complete functional visual recovery. Applying supportive operational parameters, such as the Caine criteria, aids early clinical recognition and prompt empirical thiamine administration, which can facilitate optimal visual recovery.

A critical clinical pitfall illustrated by this case is the administration of intravenous carbohydrates prior to thiamine repletion. During her initial evaluation, our patient inadvertently received intravenous 5% dextrose without concurrent thiamine for 48 hours. Because thiamine is an essential coenzyme for carbohydrate metabolism, a sudden influx of glucose can rapidly consume the body’s marginal thiamine stores. This metabolic mismatch can precipitate or exacerbate neurological decline in WE [1,2,11]. The Royal College of Obstetricians and Gynaecologists (RCOG) Green-top Guideline No. 69 explicitly warns against this and recommends that thiamine be administered prior to dextrose infusions to prevent iatrogenic WE [23]. Once WE was recognized, our patient was immediately transitioned to an evidence-based acute regimen of high-dose intravenous thiamine (500 mg thrice daily for two days, followed by 200 mg thrice daily for three days). This intensive dosing aligns with high-dose neurological and internal medicine treatment protocols for established WE [10,14]. Following recovery and completion of the acute intravenous phase, she was transitioned to a gradual oral taper. While RCOG guidelines recommend an ongoing prophylactic dose of 100 mg thrice daily for women at risk of deficiency [23], our institutional practice utilized a gradual weekly tapering strategy down to a final maintenance dose of 10 mg daily. This empirical step-down approach was safely implemented because the patient’s severe hyperemesis had resolved, allowing her to resume adequate oral nutrition. The 10 mg daily maintenance dose exceeds the standard physiological requirement during pregnancy (approximately 1.2 mg daily [11]) and served as a protective baseline against recurrence for the remainder of her gestation.

While imaging supports the diagnosis, normal imaging does not rule out WE, as demonstrated by Rees et al. (1997) and Divya et al. (2022) [1,2]. In our patient, a plain CT scan of the brain was chosen as an initial rapid imaging modality to rule out life-threatening brain pathology. The CT scan showed no abnormalities, which is common, as CT tends to be less sensitive than MRI in detecting early signs of WE [12,13]. Although MRI remains the preferred neuroimaging modality, typically showing symmetrical T2-weighted and fluid-attenuated inversion recovery (FLAIR) hyperintensities in the medial thalami, mammillary bodies, tectal plate, and periaqueductal gray matter [12,13], empirical treatment must never be delayed pending MRI availability. The absence of MRI confirmation limits definitive diagnostic certainty, rendering this a clinical and therapeutic diagnosis supported by prolonged vomiting, severe dietary restriction, substantial weight loss, ataxia, psychomotor abnormalities, ocular manifestations, and rapid response to parenteral thiamine. However, this scenario reflects a crucial real-world clinical tenet. While MRI is the preferred diagnostic modality, empirical treatment must never be delayed pending its availability, nor should a clinical diagnosis of WE be withheld when facility limitations preclude it.

Prompt recognition and empirical treatment are necessary because delayed treatment may result in irreversible maternal neurological complications and severe adverse pregnancy outcomes, including intrauterine fetal demise [3,6,10,12]. Practitioners must employ a proactive approach, placing thiamine deficiency high in the differential diagnosis for any pregnant woman exhibiting neurological symptoms following hyperemesis [3].

A limitation of our case report is the absence of formal visual field (VF) testing and macular ganglion cell layer (GCL) analysis. While the patient demonstrated excellent recovery of central Snellen visual acuity, correlating these structural OCT findings with functional VF outcomes and localized GCL measurements would provide a more comprehensive understanding of persistent subclinical deficits. The lack of neuroimaging with MRI and pretreatment blood thiamine or alternative nutritional biomarkers, such as whole-blood thiamine diphosphate, constitutes diagnostic limitations. While MRI supports the diagnosis of acute WE and baseline thiamine levels provide biochemical evidence, empirical high-dose thiamine was prioritized in this emergency obstetric setting to prevent irreversible neurological sequelae. Future clinical evaluations of optic neuropathy in WE should ideally incorporate MRI, baseline biochemical assays, and longitudinal VF and GCL assessments. Finally, the lack of long-term pediatric neurodevelopmental follow-up limits our ability to assess the extended neonatal impact of this severe maternal metabolic crisis.

## Conclusions

Severe HG complicated by nutritional depletion can present with atypical neuro-ophthalmic manifestations, including profound bilateral visual loss. Because the classic triad is frequently absent, WE must be considered early, using clinical aids such as the Caine criteria to guide empirical intervention without replacing urgent specialist assessment. Frontline management requires early nutritional assessment, recognition of prolonged vomiting and significant weight loss as critical risk factors, and patient education to seek immediate evaluation for new visual or neurological symptoms. A high index of clinical suspicion should prompt empirical thiamine administration before confirmatory testing because delayed treatment increases the risk of irreversible maternal neurological complications and adverse pregnancy outcomes.
